# Post-migration living difficulties and poor mental health associated with increased interpretation bias for threat

**DOI:** 10.1177/17470218231191442

**Published:** 2023-08-11

**Authors:** Anastasia Vikhanova, Marc S Tibber, Isabelle Mareschal

**Affiliations:** 1 Department of Psychology, School of Biological and Chemical Sciences, Queen Mary University of London, London, UK; 2 Research Department of Clinical, Educational and Health Psychology, University College London, London, UK

**Keywords:** Biological motion, discrimination, migration, mental health, cognitive biases

## Abstract

Previous research has found associations between mental health difficulties and interpretation biases, including heightened interpretation of threat from neutral or ambiguous stimuli. Building on this research, we explored associations between interpretation biases (positive and negative) and three constructs that have been linked to migrant experience: mental health symptoms (Global Severity Index [GSI]), Post-Migration Living Difficulties (PMLD), and Perceived Ethnic Discrimination Questionnaire (PEDQ). Two hundred thirty students who identified as first- (*n* = 94) or second-generation ethnic minority migrants (*n* = 68), and first-generation White migrants (*n* = 68) completed measures of GSI, PEDQ, and PMLD. They also performed an interpretation bias task using Point Light Walkers (PLW), dynamic stimuli with reduced visual input that are easily perceived as humans performing an action. Five categories of PLW were used: four that clearly depicted human forms undertaking positive, neutral, negative, or ambiguous actions, and a fifth that involved scrambled animations with no clear action or form. Participants were asked to imagine their interaction with the stimuli and rate their friendliness (positive interpretation bias) and aggressiveness (interpretation bias for threat). We found that the three groups differed on PEDQ and PMLD, with no significant differences in GSI, and the three measured were positively correlated. Poorer mental health and increased PMLD were associated with a heightened interpretation for threat of scrambled animations only. These findings have implications for understanding of the role of threat biases in mental health and the migrant experience.

## Introduction

Interpretation biases, or the tendency to interpret ambiguous information as negative or positive (Beard & Amir, 2008), represent a subset of the wider phenomena of cognitive biases, which are defined as “cases in which human cognition reliably produces representations that are systematically distorted compared to some aspects of objective reality” (Haselton et al., 2015, p. 968). Commonly, healthy participants with no underlying mental health conditions are found to exhibit a positivity bias for interpreting novel information as positive (A. Schick et al., 2013). Within clinical contexts, negative cognitive biases have been shown to play a key role in the formation and maintenance of many common mental health disorders, including depression (Gotlib et al., 2004), anxiety (Mogg et al., 1992), and post-traumatic stress disorder (PTSD) (Fani et al., 2012). Studies using ambiguous written or pictorial scenarios, scrambled sentences, or morphed emotional faces have been used to measure interpretation biases within the context of common mental health disorders (Hirsch et al., 2016), and meta-analyses have shown associations between negative interpretation biases and symptoms of depression (Everaert et al., 2017) and social anxiety (Chen et al., 2020) in the medium to large effect size range.

Whereas negative interpretation biases in depression are often investigated using negatively valenced stimuli such as sad faces or scenarios with outcomes of failure (e.g., “I will not succeed in life”), there is a large body of research, particularly in anxiety, which examines threat valenced interpretation biases from diverse stimuli, e.g., perceiving someone waving as someone wanting to hit you (Amir et al., 2005). Recently emerging evidence, however, suggests that negative (threat) and positive (benign) interpretation biases might not be the two sides of the same coin and are rather distinct constructs (Steinman et al., 2020). These authors argued that interpretation biases are often not measured through independent ratings for benign and threat interpretations, but rather as a relative score or a ratio of negative to positive biases, hence, masking one or the other. In this article, we sought to explore positive interpretation bias and interpretation bias for threat separately.

Notably, over 80% of previous research into interpretation biases (and cognitive biases more broadly) has involved participants from Western, Educated, Industrialized, Rich, and Democratic (WEIRD) societies, despite these only representing 12% of the world’s population (Henrich et al., 2010). There have been recent calls to include minoritised groups and widen research to include broader cross-cultural samples including ethnic minorities and migrant groups. One such study that addressed cross-cultural differences in interpretation biases focused on six groups of native and migrant British and Chinese participants in the United Kingdom and Hong Kong: native British, native Chinese, long- and short-term migrants to the United Kingdom and Hong Kong (Yiend et al., 2019). Participants completed a Scrambled Sentences Task, whereby they were presented with a series of six words, five of which they needed to re-arrange into a sentence under a high cognitive load (remembering strings of digits). The unused word would represent a positive or negative bias, such as “I am a born *winner* (or *loser)*” (Wenzlaff & Bates, 1998). The authors found that native British participants made fewer positive interpretations (unscrambled fewer sentences positively) compared with native Asian participants. However, migrating from the United Kingdom to Hong Kong led to an increase in these positive interpretations, whereas Chinese migrants to the United Kingdom saw a reduction in positive unscrambling of sentences. In other words, migrant participants culturally adapted to biases of the hosting country. It is important to note, though, that the authors did not measure mental health or any other parameters in their study as the focus was to establish a baseline of biases in previously untested migrant populations.

Migrants could be a population of interest in investigating cognitive biases for several reasons. First, migration is commonly associated with an increased risk of developing a range of mental health disorders, including anxiety, depression, and PTSD (Bustamante et al., 2018; Close et al., 2016; J. Lindert et al., 2009). Commonly, increased levels of mental health disorders in migrants have been attributed to Post-Migration Living Difficulties (PMLD) (Silove et al., 1997), such as difficulties in meeting basic needs (e.g., permission to work, money, access to benefits), integration issues (e.g., isolation, language problems, missing family), and ethnic discrimination. In turn, ethnic discrimination has been widely linked to mental health difficulties, including generalised and social anxiety (Levine et al., 2014; Rippy & Newman, 2006; Soto et al., 2011) and depression (Alvarez-Galvez & Rojas-Garcia, 2019; Hudson et al., 2016; Noh & Kaspar, 2003) in both migrant and non-migrant ethnic groups (Jurado et al., 2017).

Second, discrimination might make individuals more prone to misinterpreting threat in their environment. While an interpretation bias towards threat may represent an adaptive response to historical experiences of attack or discrimination, when chronically activated, it may drive or perpetuate a range of common mental health difficulties, e.g., anxiety (Craske et al., 2011; Shankman et al., 2013). Dovidio (2001) proposed that in the presence of attributionally ambiguous behaviours, such as members of the majority group choosing not to sit next to a minority on a bus, an individual from an ethnic minority is faced with a cognitively demanding task of disambiguation (van den Bos & Lind, 2002), in which they must decide whether this behaviour is driven by racial bias or some other factor (Ozier et al., 2019). Interestingly, motivations and misinterpretation of threat of a majority group in this example have been previously explored within a framework of in-group and out-group biases, i.e., favouritism towards members of one’s own group and prejudice towards members of the out-group (Brewer, 1979), who are typically perceived as threatening. For example, studies explored White participants’ negative attitudes—including enhanced perception of threat—towards ethnic minorities (Riek et al., 2006; Rios et al., 2018), or country citizens’ and “earlier” migrants’ discriminatory behaviours towards “new” migrants (Stansfield & Stone, 2018; Van der Zwan et al., 2017). However, this framework has never been utilised to investigate how prejudice and discrimination affect the *minority* group.

Taken together, these findings suggest that under conditions of uncertainty such as during the disambiguation of people’s intentions or actions, minority groups may rely on heuristics or cognitive biases, which lead to situations or cues being interpreted as more threatening than they actually are. Furthermore, such biases may be driven by experiences of adversity that are central to many migrants’ experiences. However, it remains to be examined whether such an elevation in interpretation biases for threat is associated with increased mental health difficulties, PMLD, and the experience of ethnic discrimination.

Much of the previous research into interpretation biases has relied on verbal methodologies, which may be problematic because some participants, especially migrants, may have poorer English skills than native adult speakers (Hirsch et al., 2016). Furthermore, some cognitive bias tasks show low reliability when translated from one language to another (Smith et al., 2018), which further limits current available tools for measuring interpretation biases in non-WEIRD populations. One innovative method to study interpretation biases that bypasses the need for verbal fluency is the use of biological motion stimuli or Point Light Walkers (PLW). Originally developed by Johansson (1973), PLW are an array of light dots that represent major joints, the head, and limbs of an actor’s body. Previous research has found that although limited in visual information, PLW contain sufficient key visual information for healthy participants to recognise the sex (Alaerts et al., 2011; Brooks et al., 2008), actions (Vanrie & Verfaillie, 2004), and even affective state and emotions (Atkinson et al., 2004; Clarke et al., 2005; Heberlein et al., 2004) of the walker. Importantly, unlike other types of pictorial stimuli such as emotional faces, PLW are stripped of their ethnicity, as well as any other social or contextually meaningful information.

PLW have also been used in clinical populations, particularly in those thought to have deficits in social cognition and disordered social and/or emotion processing such as schizophrenia (Okruszek & Pilecka, 2017), autism (Pavlova, 2012), and depression and anxiety. For example, Loi and colleagues (2013) found that compared with healthy controls, participants with unipolar depression struggled with recognising happy emotions from PLW stimuli. PLW have also been used to measure a type of interpretation bias called facing-the-viewer bias. In these studies, PLW are ambiguous in terms of the direction they are moving in (either walking away or towards the viewer) and have been used to index participant’s sensitivity to threat-relevant information (Heenan et al., 2014). This bias has been investigated among anxious participants, although the findings of these studies are mixed, with some reporting anxious participants exhibiting a bias towards perceiving PLW as facing towards them (Yiltiz & Chen, 2018), and some reporting the opposite effect (Van de Cruys et al., 2013). Both types of effects have been conceptually linked to the interpretation of threat, with a PLW walking away indicating a “wishful thinking” bias (for avoidance of an interaction or a threat), and PLW walking towards as a negative interpretation bias for threat.

Recently, PLW have been used to investigate threat perception directly. For example, Satchell et al. (2021) presented healthy participants with static and moving (PLW) images of 23 individuals who previously self-reported high levels of aggressiveness. They found that PLW (especially male figures) were rated as more aggressive compared with static images, and healthy participants were generally accurate at recognising threat from PLW. To our knowledge, no other studies have used PLW as a way of probing interpretation biases for threat, including those in migrant groups. Therefore, given the accuracy of healthy participants at recognising emotion from PLW, PLW’s previous use to measure emotion recognition deficits and to elicit interpretation biases in clinical populations, we adapted PLW to measure interpretation bias (both positive and for threat) as an innovative non-verbal, contextually and ethically neutral task.

Building on previous research into the association between adverse life experiences, mental health difficulties, and threat interpretation biases in migrant populations, we sought to explore the relationship between these factors in an undergraduate student migrant population. Although interpretation biases have previously been explored in migrant populations (Yiend et al., 2019), these studies did not investigate interpretation biases for threat more specifically (only negative vs. positive) and further did not include measures of mental health or other variables relevant to the migrant experience. We selected several UK-based migrant groups in line with previous reports of differences in the effects of ethnic discrimination on mental health that are dependent on generational status. For example, second-generation migrants, defined as people who were born and reside in a country that at least one of their parents previously entered as a migrant, appear to be affected more by ethnic discrimination than first-generation migrants who were born outside the country (Giuliani et al., 2018; Yazdiha, 2019). At the same time, much of this research has confounded ethnicity and migration (A. Lindert, Korzilius, et al., 2008). For example, White migrants from Eastern Europe may also experience PMLD (Madden et al., 2017), and further, may experience discrimination in the United Kingdom due to their foreign accents (Fernández-Reino, 2020).

Thus, in this study, we explored three participant groups: first- and second-generation ethnic minority migrants and White first-generation migrants from non–English-speaking countries, allowing us to directly compare findings across these groups, while avoiding confounding of ethnicity and migration status. In line with previous findings (Bustamante et al., 2018; Fernández-Reino, 2020; Giuliani et al., 2018; Steel et al., 1999), we hypothesised the following:

*H1.* First- and second-generation ethnic minority migrant students and first-generation White migrant students would differ in their experiences of PMLD, perceived ethnic discrimination, and mental health difficulties.

Specifically, given inconsistencies in previous findings, we tentatively predicted a gradient in mental health, PMLD, and discrimination scores running from high to low across first-generation ethnic minority, second-generation ethnic minority, and first-generation White groups. We also expected the following:

*H2*. All three measures (mental health, discrimination, and PMLD) would be positively correlated across groups.

Finally, with respect to interpretation biases, we predicted the following:

*H3*. Participants with poorer mental health, higher perceived ethnic discrimination, and higher PMLD scores would display interpretation biases, rating all types of PLW stimuli (positive, negative, neutral, and ambiguous) as lower on friendliness (low positive interpretation bias) and higher on aggressiveness scales (high interpretation bias for threat).

## Method

The study was approved by the ethics board of Queen Mary University of London (QMERC2019/70) and participants gave written informed consent to take part. Participants were recruited through advertisement on campus and received course credit or £7 for their participation. This study was not pre-registered.

### Self-report measures

Participants provided basic demographic information including age and gender, in addition to information about existing mental health diagnoses, access to mental health treatment, and length of stay in the United Kingdom. Participants were also asked about their subjective social status, which was measured by the MacArthur Scale of Subjective Social Status (Adler et al., 2000). Following this, participants completed the following three questionnaires in a randomised order.

#### Brief Symptom Inventory

To measure mental health symptoms, the Brief Symptom Inventory (BSI) was used (Derogatis & Spencer, 1993). BSI is a 53-item measure with a five-point scale ranging from 0 (“not at all”) to 4 (“extremely”) that aims to assess how much a person has been affected by certain symptoms in the past 7 days. It consists of nine subscales measuring primary symptom dimensions of somatisation, obsession–compulsion, interpersonal sensitivity, depression, anxiety, hostility, phobic anxiety, paranoid ideation, and psychoticism. This scale has been widely used for both clinical and non-clinical populations and has previously been validated and commonly used in student groups to assess psychological distress (Cochran & Daniel Hale, 1985; Hayes, 1997; Sher et al., 1996). The BSI also includes three indices of global distress: Global Severity Index (GSI, measures current level of symptomatology), Positive Symptom Distress Index (intensity of symptoms), and Positive Symptom Total (number of reported symptoms), which show good reliability and validity (Derogatis & Spencer, 1993). In this study, we report the GSI only, which is calculated as an average of scores on all 53 items. This is because we were not interested in linking specific disorders or their intensity to interpretation biases, but rather exploring the association between the overall level of current mental health and biases. In this study, Cronbach’s alpha was .97 indicating high internal consistency.

#### Perceived Ethnic Discrimination Questionnaire

We used the Perceived Ethnic Discrimination Questionnaire (PEDQ) to measure discrimination (Contrada et al., 2001). This is a 22-item measure with a seven-point Likert-type scale measuring the frequency of different discrimination events taking place in the past 3 months, which ranged from 1 (“never”) to 7 (“very often”). It consists of seven subscales: verbal rejection, avoidance, exclusion, denial of equal treatment, devaluating action, threat of violence, and aggression, although a total score was used for the purpose of this study. Scores range from 22 to 154, with higher scores indicating more experiences of ethnic discrimination/racism. This tool was selected due to its high validity and good reliability in students and across different ethnic groups. Thus, many other tools have only been validated in the United States or are only appropriate for specific ethnicities (Atkins, 2014). The authors reported good internal consistency (Cronbach’s alpha = .81), and good construct validity using inverse correlations with measures of mental health and prejudice (Contrada et al., 2001). In this study, Cronbach’s alpha was .93 indicating high internal consistency.

#### PMLD checklist

We used the PMLD checklist to measure the severity index of issues students might have experienced as a result of being from a migrant group (Silove et al., 1997). The checklist includes 22 items of the most common issues refugees and migrants might experience in a host country, covering areas such as “meeting basic needs,” “health care,” “relationships,” “integration difficulties,” and “housing problems,” although a total score was used for the purpose of this study. It is important to note that “discrimination” is also included in the checklist as one of the items (the removal of this item did not result in any change of our findings as discussed in the “Results” section). Participants rated items on a scale from 0 (“does not affect me”) to 5 (“very serious problem”) and had the option of adding up to four of their own items to the list and subsequently rating them as a standard part of the questionnaire as designed by Silove et al. (1997). This is to ensure it captures all PMLD that may be affecting a participant. In refugee populations, the scale is typically used to measure/count the number of severe and very severe issues migrants experience (Steel et al., 1999). However, the authors of the scale highlighted that the scoring of the questionnaire can be modified depending on the migrant group of interest, e.g., economic migrants would unlikely experience these severity types of issues. Because our group of interest was student migrants among whom we expected a less severe range of adverse experiences, we calculated a total number of problems that were rated as “moderate” or above. These adaptations are often made depending on the participant group of interest and especially location and cultural context (e.g., M. Schick et al., 2016, 2018; Spiller et al., 2016).

### PLW stimuli selection

In line with common interpretation bias designs (see Hirsch et al., 2016 for a review), we have chosen to include positive, negative, neutral, and ambiguous categories of PLW in this study. To identify PLW corresponding to our four categories of action (positive, negative, neutral, and ambiguous), 98 PLW animations were selected from a dataset of 500, obtained online or by contacting the authors directly (Alaerts et al., 2011; Heberlein et al., 2004; Lapenta et al., 2017; Manera et al., 2010; Shipley & Brumberg, 2005; Vanrie & Verfaillie, 2004). These stimuli were then piloted with 33 undergraduate students (21 female, age range: 19–32 [*M* = 25.06, *SD* = 2.57]), who were asked to rate, for each stimulus, whether it was positive, negative, or neutral, and how ambiguous it seemed on a scale 0 (not at all ambiguous) to 100 (extremely ambiguous). Based on the responses, a subset of 35 stimuli were used in the final study: five animations in each of the positive, negative, and neutral action categories, and 15 animations in the ambiguous action category (see Supplementary Materials for choice selection). A further five scrambled animations comprising randomly moving dots that are not representative of any human form or action and expected to be perceived as neither aggressive nor friendly were also included. Scrambled animations and the procedure for their generation can be found in Alaerts et al. (2011).

### Testing protocol

Testing took part in two separate sessions. First, participants received an online link to a consent form and questionnaires 24-hr prior to the lab-based component of the study and were completed beforehand. Second, as part of a larger project, participants completed three computerised tasks, including the interpretation bias task reported here. Testing was completed in a dimly lit cubicle with the researcher present, who gave verbal instructions and ensured participants’ understanding of the task.

The PLW stimuli were presented one at a time in a randomised order via Qualtrics on a Dell PC on a screen size of 44 × 25 cm (1600 × 900 pixels). At the viewing distance of 57 cm, one pixel subtended 1.62 arcmin. The original PLW stimuli were of different sizes so were standardised to the same height at presentation (6.66° of visual angle) while maintaining their original aspect ratios. The stimuli across all PLW types randomly varied in their duration of action completion (between 1 and 12 s) and so were played on a continuous loop until participants responded. Participants were asked to imagine having a social interaction with the PLW and subsequently rate how (1) friendly and (2) aggressive the PLW were towards them using a Likert-type scale from 0 (“not at all”) to 3 (“very”). Participants were also asked an additional question as to whether they would find this social interaction pleasant or whether they would try to avoid it. However, due to an error in phrasing of the question (pleasantness and avoidance were not rated separately), results of this question are not reported here.

At the end of the study, participants were given a debrief sheet with information about the study, aims, and predictions for the experiments along with details of services available both inside and outside the university for mental health support.

### Data analysis

In this study, we have measured a bias as a raw score of a degree to which participants saw PLW as friendly (or positive, 0–3) or aggressive (interpretation bias for threat, 0–3), with higher scores indicating higher bias. In line with recent research (Schmitt et al., 2021; Steinman et al., 2020), we chose to not calculate interpretation bias as the proportion of one score to the other, or by subtracting one from the other, as has been commonly done in previous research (Hirsch et al., 2016), as this might have concealed a true bias in either direction. Thus, participants could have exhibited both positive and negative bias for each PLW. This was crucial for our category of ambiguous stimuli (that were open to interpretation, be that positive or for threat), as we anticipated positive PLW to be rated as highly friendly and not at all aggressive, opposite for negative PLW, and neutral PLW were expected to receive a score close to 0 on both scales for an “unbiased” result.

An a priori power analysis was conducted using G*Power3 (Faul et al., 2007) to calculate the minimum sample size for a two-tailed correlation between measures of interest and interpretation biases, assuming a medium effect size (*r* = .40) at an alpha of .05 and power of 0.95. A medium effect size was selected based on previous studies on the relationship between mental health and interpretation biases, which have reported medium to strong effect sizes. Results indicated a minimum sample of 75 participants; however, as this experiment was part of a larger study that focused on group differences on a set of three cognitive bias tasks, we tripled this (minimum *n* = 225).

Data analysis was completed using SPSS v.28 (International Business Machines Corporation, 2021). Considering our first hypothesis regarding group differences on PEDQ, PMLD, and GSI measures, we ran an analysis of variance (ANOVA) or a Kruskal–Wallis test when the assumption of equal variance was not met. Post hoc Bonferroni-corrected (alpha = .02) follow-up independent samples *t* tests and Mann–Whitney tests were run to explore further differences between first- and second-generation ethnic minority and White participants.

For the second hypothesis regarding the relationship between the three measures (GSI, PEDQ, and PMLD checklist), separate Spearman’s bivariate correlations with bias-corrected and accelerated (BCa) bootstrap interval procedure (1,000 repeats) were run and Bonferroni corrected for three comparisons (corrected alpha = .016). The same procedure was implemented for the third hypothesis regarding the relationship between interpretation biases (as measured using friendliness [positive interpretation bias] and aggressiveness ratings [interpretation bias for threat] on the PLW) and our three measures of interest (GSI, PEDQ, and PMLD checklist). Bonferroni corrections were made for 10 multiple comparisons (corrected alpha = .005) reflecting two types of measures (friendliness and aggressiveness) and five types of PLW stimuli analysed in relation to each measure of interest (GSI, PEDQ, and PMLD checklist). Bayes factors were calculated following the procedure described in Heo et al. (2020) using JASP software (JASP Team, 2020).

## Results

### Participants

A total of 230 participants (171 females; 58 males; 1 preferred not to say) aged 18–33 years (*M* = 21.26, *SD* = 3.32) took part in the study. None of the participants were excluded from data analysis. The participants were from three groups: first-generation ethnic minority migrants (*n* = 94), second-generation ethnic minority migrants (*n* = 68), and first-generation White migrants (*n* = 68), all of whom were students. The ethnic minority participants were from diverse ethnic groups (see Table S2 in Supplementary Materials). Regarding religion, Islam was the most reported religion with 35% of participants identifying as Muslim (68% of second-generation, 34% of first-generation and 3% of White participants), followed by 24% reporting having no religion (46% White, 21% first generation and 7% second generation) and 23% of Christians (44% White, 16% first generation and 12% second generation). Further 11% reported Hindu as their religion (21% first generation and 9% second generation).

There were group differences in the time spent in the United Kingdom (Table 1), with second-generation ethnic minorities having spent their whole life in the country. A Bonferroni-corrected (corrected alpha = .02) independent *t* test revealed no significant differences with respect to the number of years in the United Kingdom between two first-generation migrant groups (*p* = .032). Regarding perceived social status, the only significant difference after Bonferroni correction was between White first-generation group who scored higher than the second-generation ethnic minority group, *t*(134) = 2.40, *p* = .018, *d* = 0.41. Across groups, students reported their social status on a ladder (ranging 1–10) just above the middle range (*M* = 5.83, Table 1). There were no further group or gender differences found.

**Table 1.** Participants’ characteristics.

	First-generation migrants (White)	First-generation ethnic minority migrants	Second-generation ethnic minority migrants
*N*	68	94	68
Gender	21% male	36% male	15% male
Age, *M* (*SD*)	21.74 (3.53)	22.27 (3.74)	19.38 (1.32)
Time spent in the United Kingdom (years), *M* (*SD*)	3.54 (3.60)	5.41 (6.45)	19.02 (3.08)
Social status, *M* (*SD*)	6.06 (1.41)	5.95 (1.42)	5.47 (1.45)
GSI, *M* (*SD*)	1.03 (0.68)	1.00 (0.76)	0.98 (0.72)
PEDQ, *M* (*SD*)	33.72 (13.06)	42.63 (20.08)	44.59 (20.18)
PMLD, *M* (*SD*)	3.94 (3.87)	7.26 (7.23)	3.44 (5.42)

GSI: Global Severity Index (measure of mental health); PEDQ: Perceived Ethnic Discrimination Questionnaire; PMLD: Post-Migration Living Difficulties.

### Mental health, perceived ethnic discrimination, and PMLD

To examine group differences between first- and second-generation ethnic minority and White migrant participants in mental health (GSI), ethnic discrimination (PEDQ), and PMLD (checklist), we ran an ANOVA on GSI for which the assumption of equal variances was met, *F*(2, 227) = 0.78, *p* = .459. Levene’s test indicated unequal variances for PEDQ, *F*(2, 227) = 8.69, *p* < .001, and PMLD checklist, *F*(2, 227) = 22.34, *p* < .001, thus Kruskal–Wallis tests were used for these instead.

The three groups did not differ significantly with respect to GSI (*p* = .913) (Table 1) but did differ significantly on PEDQ, χ^2^(2) = 13.11, *p* = .001. Consistent with the first hypothesis, post hoc Bonferroni-corrected Mann–Whitney tests revealed that White participants’ PEDQ scores were lower than those of first-generation ethnic minority migrants (*U* = 2422.50, *p* = .009) and those of second-generation ethnic minority migrants (*U* = 1477, *p* < .001). However, contrary to H1, first- and second-generation ethnic minority groups did not differ in their PEDQ scores (*p* = .427).

With respect to scores on the PMLD checklist, a main effect of group was also found, χ^2^(2) = 13.14, *p* = .001. Post hoc Bonferroni-corrected Mann–Whitney tests showed that first-generation ethnic minority migrants experienced significantly more PMLD than second-generation migrants (*U* = 2247.50, *p* = .001), but not more than White migrants (*p* = .052). Second-generation and White migrants did not differ following a post hoc Bonferroni correction (*p* = .018), contrary to the first hypothesis.

**Figure 1.**
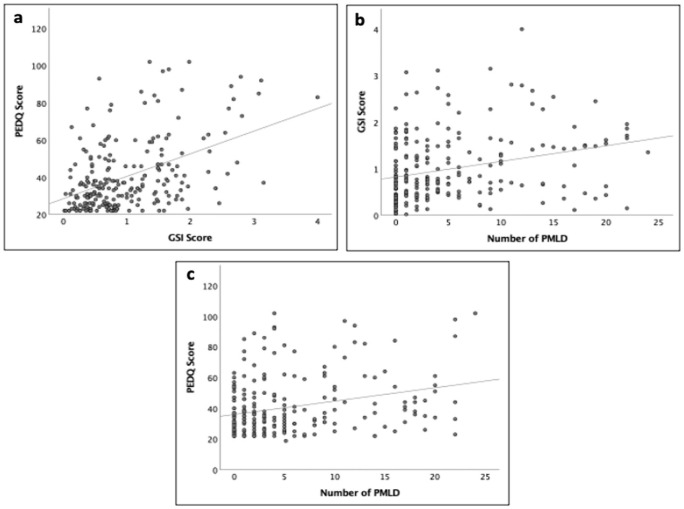
Scatter plots of (a) the correlation between ethnic discrimination (PEDQ) and mental health (GSI) scores; (b) the correlation between mental health (GSI) scores and the number of Post-Migration Living Difficulties (PMLD); (c) the correlation between ethnic discrimination (PEDQ) scores and the number of Post-Migration Living Difficulties (PMLD). Data are pooled across the three participant groups. GSI: Global Severity Index (measure of mental health); PEDQ: Perceived Ethnic Discrimination Questionnaire.

Finally, and in line with our second hypothesis about the positive relationship between the three measures, we found significant positive correlations between all variables, i.e., those scoring higher on any one measure also scored higher on the other two. Thus, mental health scores were positively correlated with discrimination, *r*(228) = .42, *p* < . 001, 95% confidence interval (CI) = [0.30, 0.51], *BF_10_* = 9.89 × 10^+10^ and PMLD, *r*(228) = .32, *p* < . 001, 95% CI = [0.19, 0.45], *BF_10_* = 1,073.37. Discrimination was also positively correlated with PMLD, *r*(228) = .29, *p* < . 001, 95% CI = [0.16, 0.40], *BF_10_* = 981.41 (Figure 1).

### Associations with interpretation biases

Participant groups (first-generation ethnic minority, second-generation ethnic minority, and first-generation White) did not differ with respect to their mean ratings of friendliness or aggressiveness for any of the five types of PLW (see Supplementary Materials). Consequently, we report analyses on data pooled across participant groups.

Regarding our third hypothesis, no significant correlations were found between our three measures of interest (GSI, PEDQ, and PMLD checklist) and friendliness ratings for scrambled, neutral, positive, negative, or ambiguous PLW (Table 2). However, significant correlations were found between GSI and aggressiveness ratings (*r* = .24, *p* < .001, *BF_10_* = 9.91), as well as PMLD and aggressiveness ratings, for *scrambled* animations (*r* = .23, *p* < .001, *BF_10_* = 13.17), and these survived correction for multiple comparisons (corrected alpha = .001). Thus, those who scored higher on GSI and PMLD checklist rated scrambled stimuli as more aggressive. There were also curious associations between GSI and aggressiveness rating for *ambiguous* stimuli (*r* = .14, *p* = .029), as well as between PEDQ and aggressiveness for *neutral* stimuli (*r* = .13, *p* = .045), although these did not remain significant following Bonferroni correction, or according to the Bayes factor (Table 2).

**Table 2.** Correlations between stimuli ratings (friendliness/aggressiveness) and three measures of interest (GSI, PEDQ, and PMLD checklist).

Stimulus type	Rating	GSI				PEDQ				PMLD			
		*r*	*p* value	95% CI	*BF_10_*	*r*	*p* value	95% CI	*BF_10_*	*r*	*p* value	95% CI	*BF_10_*
Scrambled	Friendliness	−.06	.392	[−0.19, 0.07]	0.09	−.07	.489	[−0.17, 0.09]	0.08	−.05	.501	[−0.18, 0.10]	0.10
	Aggressiveness	.24**	.001	[0.11, 0.35]	9.91	.12	.070	[−0.01, 0.25]	0.57	.23	.001**	[0.10, 0.36]	13.17
Positive	Friendliness	.06	.338	[−0.09, 0.20]	0.10	−.02	.744	[−0.16, 0.13]	0.09	.08	.256	[−0.06, 0.20]	0.11
	Aggressiveness	.08	.230	[−0.04, 0.20]	0.22	.12	.074	[−0.01, 0.24]	0.22	−.01	.965	[−0.14, 0.14]	0.29
Neutral	Friendliness	.01	.847	[−0.13, 0.15]	0.11	.06	.397	[−0.09, 0.20]	0.17	.06	.361	[−0.10, 0.22]	0.20
	Aggressiveness	.10	.143	[−0.04, 0.22]	0.43	.13*	.045	[0.01, 0.25]	0.39	.05	.436	[−0.09, 0.18]	0.49
Negative	Friendliness	.10	.133	[−0.04, 0.23]	0.18	−.03	.642	[−0.16, 0.11]	0.08	.09	.164	[−0.06, 0.23]	0.44
	Aggressiveness	.04	.537	[−0.08, 0.17]	0.09	−.01	.987	[−0.14, 0.13]	0.10	.05	.493	[−0.09, 0.18]	0.08
Ambiguous	Friendliness	.11	.108	[−0.03, 0.24]	0.14	−.01	.859	[−0.14, 0.12]	0.12	.06	.403	[−0.08, 0.19]	0.14
	Aggressiveness	.14*	.029	[0.01, 0.28]	0.88	.09	.168	[−0.05, 0.22]	0.34	.07	.288	[−0.06, 0.20]	0.20

GSI: Global Severity Index; PEDQ: Perceived Ethnic Discrimination Questionnaire; PMLD: Post-Migration Living Difficulties.

* *p* < .05. **p < .005 (remaining significant following correction for 10 comparisons).

## Discussion

The aim of this study was twofold. First, we sought to explore PMLD, ethnic discrimination, and mental health differences in three groups: first-generation White and ethnic minority groups and second-generation ethnic minority group, as well as the relationship between the three factors of interest. Here, we found that PMLD checklist scores were highest for first-generation ethnic minority migrants, and PEDQ scores were higher for both first- and second-generation ethnic migrants than first-generation White migrants. However, there were no differences in GSI scores between the three groups. In line with the second hypothesis, we found a positive association between all three factors of interest (GSI, PMLD, and PEDQ), with groups scoring higher on one measure also scoring higher on the other measures. Second, we sought to investigate the relationship between PMLD, discrimination, and mental health and two types of interpretation biases, positive interpretation bias and interpretation bias for threat. We found that neither bias (i.e., neither friendliness not aggressiveness ratings) for the four PLW types that depicted human movement (positive, negative, neutral, and ambiguous) correlated with measures of mental health, PMLD, or ethnic discrimination. However, significant positive associations were found between interpretation bias for threat (aggressiveness ratings) of scrambled PLW with mental health and PMLD checklist scores, although not PEDQ.

In our data, first-generation ethnic minority migrants experienced the highest number of PMLD relative to White migrants and to second-generation ethnic minority migrants, with no difference between the latter two groups. Previous studies exploring the differences between first- and second-generation migrants have tended to focus on PMLD in relation to mental health only (e.g., Usama et al., 2021) or else explored a wider range of migration difficulties in the two groups separately (Aragona et al., 2012; Gomula & Koziel, 2015). Furthermore, these studies have traditionally excluded White migrants completely. Our finding that White migrant participants report similar numbers of PMLD to second-generation ethnic minority migrants warrants further exploration, particularly in light of the experiences and challenges faced by EU migrants in the United Kingdom following Brexit and the reported resultant rise in racism and discrimination (Virdee & McGeever, 2018).

Our findings suggest that PMLD, although found to be less common in our student migrant samples than in other populations of immigrants (Aragona et al., 2012), still have a significant relationship with migrant students’ mental health, although the cross-sectional design of the study precludes inferences about the underlying direction of causality. However, in our study, there were no differences in reported mental health symptoms between the three groups included. This is at odds with previous literature that found that first-generation economic migrants were at a higher risk of depression, as a result of more post-migration and integration difficulties and discrimination, regardless of their ethnicity (Levecque & Van Rossem, 2015). One explanation for this discrepancy is that participants in this study were students, including international students, who might have a higher socioeconomic status (SES) and educational attainment than economic migrants investigated in earlier studies (Goodwin et al., 2018; J. Lindert, Schouler-Ocak, et al., 2008). In turn, higher SES has been linked to better mental health (Dohrenwend, 1990), and greater likelihood of seeking mental health support (Steele et al., 2007). Moreover, students have easier access to mental health services within the university, while (non-student) economic migrants receive support through national health care systems that can be slower and/or overburdened. Therefore, it is possible that the similarity in mental health scores reflects the biased nature of our sample, i.e., higher SES and educational attainment, which might offer greater protection against stressors.

Finally, we found no group differences in ethnic discrimination scores between first- and second-generation migrants, contrary to our predictions and several previous reports suggesting that second-generation migrants perceive more ethnic discrimination and are more psychologically affected by it (Giuliani et al., 2018; Yazdiha, 2019). General scores obtained on the PEDQ were lower than other previously reported community samples, e.g., Brondolo et al. (2008), and although the measure included a wide variety of probable discrimination settings, certain questions might have been less relevant for student populations, such as those related to workplaces or owned property. Moreover, a lack of differences between first- and second-generation ethnic migrants may also reflect the nature of the university where the study was conducted. Queen Mary University of London is very ethnically diverse, and it is possible that our students’ experiences might be different from those of the general migrant population, or perhaps even students studying in less diverse universities or cities in the United Kingdom. In support of this, previous studies in the United States have found that the effects of discrimination on ethnic minority students’ well-being could be especially pronounced for those attending predominantly White universities (Neville et al., 2004).

Finally, the second aim in this study was to explore associations between interpretation biases (positive and for threat), mental health, PMLD, and perceived ethnic discrimination. We expected that the ratings for positive, negative, neutral, and ambiguous PLW would have a relationship with the aforementioned measures, such that positive interpretation bias (friendliness ratings) and interpretation bias for threat (aggressiveness ratings) would be correlated with mental health, PMLD, and ethnic discrimination scores. In other words, we expected increased scores on the three measures to be associated with interpretation bias for threat and vice versa. PLW have not previously been used in this context, but there is a large body of research linking increased perception of threat in ambiguous stimuli to a variety of mental health disorders (Beard & Amir, 2010; Bianchi et al., 2018; Elwood et al., 2007; Eysenck et al., 1991; Yoon & Zinbarg, 2007). We found no support for our hypothesis: neither aggressiveness nor friendliness ratings for any of the PLW types depicting coherent human actions were associated with our measures of interest. However, we did find evidence for increased interpretation bias for threat only (i.e., high aggressiveness but not friendliness ratings) with *scrambled* stimuli among individuals scoring highly for mental health difficulties, PMLD, and ethnic discrimination, although the latter did not remain significant after correction for multiple comparisons.

In terms of why the threat bias was only evident with scrambled stimuli, one possibility that is consistent with a Bayesian interpretation (Clark, 2015), is that the sought effects, in this case, bias for threat, are relatively weak and hence were only revealed under conditions of maximum uncertainty, i.e., conditions under which biases or prior expectations are most likely to be activated. Thus, scrambled stimuli contain *no* coherent actions. Nonetheless, it has been shown that healthy adults can identify emotions from *scrambled* PLW at an above-chance level based on local motion information alone (Spencer et al., 2016), and that ratings of animacy for scrambled PLW increased when the PLW animation was consistent with the direction of gravity (Thurman & Lu, 2013). The authors subsequently extended these findings to social interactions and found that participants identified social interactions in scrambled PLW at above-chance levels when stimulus motion energy was maintained (Thurman & Lu, 2014). Thus, despite their lack of *coherent action* and high uncertainty, such scrambled animations do contain information that participants interpret. In contrast, the other (coherent action) stimuli used in the study may have carried too much information (i.e., been of low uncertainty) to optimally reveal prior expectations. This is supported by the relatively modest ratings of ambiguity for these stimuli detailed in Supplementary Materials.

At the same time, it is surprising that we did not find any evidence for positive interpretation bias, neither in coherent nor in scrambled stimuli. Previous research highlighted the need to explore positive and negative interpretation biases separately (Steinman et al., 2020), particularly in light of emerging evidence that positive interpretation biases can be protective of mental health issues (e.g., Bean et al., 2023). However, there has also been some recent contradicting evidence, whereby negative interpretation bias is found to be associated with negative mental health outcomes (higher anxiety), but positive interpretation bias is not found to be associated with positive outcomes (lower anxiety) (Schmitt et al., 2021). Our results align with this, as positive interpretation bias was not associated with mental health or PEDQ/PMLD in any direction. One potential explanation for this might be the specificity of our sample in terms of gender imbalance and ethnic and religious diversity of participants and its impact on self-reported mental health difficulties. Females are usually found to have more mental health difficulties (Otten et al., 2021), while men tend to significantly underreport them (Smith et al., 2018). This is further complicated by sometimes prejudiced views on mental health, which contributes to mental health underreporting in certain ethnic minorities (McGuire & Miranda, 2008). Therefore, it might be possible that several factors have contributed to lower *reported* rates of mental health in the current sample, despite higher *true* numbers, which have precluded finding some of the associations for both positive interpretation bias and interpretation bias for threat.

With respect to its strengths, this study is the first to directly investigate the relationship between mental health, PMLD, and perceived ethnic discrimination, and to examine how these relate to positive and negative interpretation biases across three different migrant groups. By comparing first-generation ethnic, second-generation ethnic, and first-generation White migrants, we were able to explore how post-migration stressors link to mental health and cognition in these groups, while addressing existing limitations in the literature, including the fact that these groups have traditionally been studied separately (precluding direct comparisons) (Aragona et al., 2013; de Freitas et al., 2018; Ikram et al., 2015) and that EU and international White migrants have traditionally been excluded (therefore confounding migration and ethnicity) (Madden et al., 2017).

This study contributes to a large body of research documenting the negative impacts of living difficulties associated with the migrant experience (including discrimination) on mental health, as well as the links between mental health and cognition. Future research should build on this work, but also move towards an integration of the fields, exploring potential causal pathways and mechanisms underlying such links. For example, previous research has suggested that discrimination may affect adversely cognition, particularly with respect to executive functions such as inhibition, shifting, and updating (see Ozier et al., 2019 for review), and that such effects may amplify cognitive biases and heuristics that are associated with mental health (Stanovich et al., 2016). Future research, building on our own findings using a perceptual paradigm, might employ a longitudinal research design to explore whether the perceptual biases we report play a mediating role in the association between well-established adverse migrant experiences and the development of mental health difficulties.

In addition to its strengths, there are some limitations to consider in this study. First, ambiguity of coherent action stimuli was limited by the fact that our ambiguous PLW still performed a clear action unlike other types of stimuli used in interpretation bias designs. It may be that being able to identify an action, regardless of what it was, reduced the uncertainty required to elicit a perception of threat. Second, it has been suggested that mental health disorders such as anxiety are perpetuated by avoidance of threatening stimuli rather than an increased perception of threat (Kashdan et al., 2013; Trew, 2011), although the two are obviously not mutually exclusive. Therefore, in addition to measuring threat perception, it would be useful to measure avoidance of the stimuli. Finally, even though our stimuli fulfilled the selected categories of positive/negative/neutral social actions, it would be useful to control the perceived intensity and ambiguity of the action as is now possible from several new databases of PLW (Bidet-Ildei et al., 2023; Okruszek & Chrustowicz, 2020).

## Conclusion

This study is the first to directly investigate the relationship between mental health, PMLD, ethnic discrimination, and interpretation biases in first- and second-generation ethnic minority migrants and White first-generation migrants, facilitating direct comparison of parameters of interest without confounding ethnicity and migration status. We found that although the three groups did not differ on mental health measures, PMLD were higher in first-generation ethnic minority students and self-reported PEDQ scores were elevated in both ethnic minority participant groups. Furthermore, we found that individuals scoring higher on one measure were more likely to score higher on the other two measures, suggesting that discrimination, mental health, and PMLD likely co-occur, and potentially, share common etiological mechanisms. Finally, we found that PMLD and mental health difficulties were associated with increased interpretation bias for threat, although this was found for scrambled PLW stimuli only, i.e., under conditions of maximum uncertainty, that we would argue were most likely to elicit prior expectations/interpretation biases. These findings suggest that adverse life events associated with the student migrant experience may drive interpretation biases linked to poorer mental health; however, future studies are needed to elucidate the underlying mechanisms and directions of causality involved.

## Supplemental Material

sj-docx-1-qjp-10.1177_17470218231191442 – Supplemental material for Post-migration living difficulties and poor mental health associated with increased interpretation bias for threat Supplemental material, sj-docx-1-qjp-10.1177_17470218231191442 for Post-migration living difficulties and poor mental health associated with increased interpretation bias for threat by Anastasia Vikhanova, Marc S Tibber and Isabelle Mareschal in Quarterly Journal of Experimental Psychology
