# Supplementary material for: Post-migration living difficulties and poor mental health associated with increased interpretation bias for threat
Source: Q J Exp Psychol (Hove). 2023 Aug 11;77(6):1154–68. doi: 10.1177/17470218231191442 (PMC11103921; doi:10.1177/17470218231191442)
Supplement: sj-docx-1-qjp-10.1177_17470218231191442 – Supplemental material for Post-migration living difficulties and poor mental health associated with increased interpretation bias for threat [file sj-docx-1-qjp-10.1177_17470218231191442.docx]

Supplementary Material for:

**Post-Migration Living Difficulties and Poor Mental Health Associated with Increased Interpretation Bias for Threat**

Dr Anastasia Vikhanova^1a^, Dr Marc S Tibber^2^, Dr Isabelle Mareschal^1^

^1^ School of Biological and Chemical Sciences, Department of Psychology, Queen Mary University of London, London, UK, E1 4NS

^2^ Research Department of Clinical, Educational and Health Psychology, UCL, London, UK, WC1E 6DH

^a^ Corresponding author, [a.vikhanova@qmul.ac.uk](mailto:a.vikhanova@qmul.ac.uk)

**Pilot study**

A pilot study was run in order to identify four categories of Point Light Walkers (PLW) stimuli: three unambiguous action categories (positive, negative and neutral action), and one ambiguous action category. Ninety-eight PLW animations were selected from a dataset of 500, obtained online or by contacting the authors (Alaerts et al., 2011; Heberlein et al., 2004; Lapenta et al., 2017; Manera et al., 2010; Shipley & Brumberg, 2005; Vanrie & Verfaillie, 2004). Scrambled images were obtained separately from Alaerts et al. (2011).

Thirty-three undergraduate students (21 female, age range 19-32 [*M*=25.06, *SD*=2.57]) who did not take part in the main experiment completed the pilot study on their personal computers. They viewed 98 individual stimuli (unlimited viewing presentation) using Qualtrics. For each stimulus, participants were asked to imagine interacting with the PLW and to rate whether they would view its action as positive, negative, or neutral towards them. They were also asked to rate the ambiguity on a scale from 0-100 of the PLW’s action and were told that if they could think of multiple descriptions of the action or no descriptions, it should be rated as ambiguous. PLW stimuli were all standardized to a height of 240 pixels whilst maintaining the aspect ratio and participants were instructed to view the stimuli at a normal viewing distance (e.g. arm’s length).

For the negative, positive and neutral (low ambiguity) PLW, we selected stimuli where (a) at least 80% of participants gave them the same rating (negative, neutral, or positive), and (b) the stimulus ambiguity ratings were lower than the mean ambiguity of all PLW (e.g. less than 34.61). For the ambiguous PLW, we selected stimuli where (a) there was less than 80% agreement between participants’ action rating (negative, neutral, or positive) and (b) the stimulus had an ambiguity rating higher than the mean ambiguity of all PLW.

The final stimulus set comprised of five images in each of the positive, negative, and neutral action categories, and 15 images in the ambiguous action category (Table S1). Following Bonferroni correction, the ambiguous stimuli were rated significantly more ambiguous than positive (*t*(4) = 6.22, *p* = .018, *d* = 4.45), negative (*t*(4) = 8.02, *p* = .006, *d* = 4.68) and neutral stimuli (*t*(4) = 7.24, *p* = .012, *d* = 5.68). There were no significant differences with respect to perceived ambiguity between positive and negative (*p* = 1), positive and neutral (*p* = 1), and negative and neutral (*p* = 1) stimuli.

**Point Light Walker ratings**

To examine PLW ratings on aggressiveness /pleasantness we carried out *post-hoc Bonferroni* corrected t-tests. There were significant differences between the ratings for all five PLW types. Scrambled (*t*(229) = 18.54, *p* < .001, *d* = 1.81), positive (*t*(229) = 56.01, *p* < .001, *d* = 5.89), and neutral (*t*(229) = 17.33, *p* < .001, *d* = 1.64) were rated as more friendly than aggressive, whereas negative stimuli (*t*(229) = 47.03, *p* < .001, *d* = 4.97) were rated as significantly more aggressive than friendly. Ambiguous stimuli did not differ in their ratings of friendliness and aggressiveness (*p* = .060). Friendliness and aggressiveness ratings differed across all types of stimuli (all at *p* < .001). Positive stimuli were rated the friendliest, followed by scrambled, neutral, ambiguous, and negative PLW. Negative stimuli were rated the most aggressive, followed by ambiguous, scrambled, neutral and positive (Figure S1).

**Table S1**

*Stimuli Description*

| Stimulus name | Emotion rating | Ambiguity rating | Original database |
| --- | --- | --- | --- |
| Positive | | | |
| CF08 | 91% positive, 9% neutral | 24.36 | Heberlein^1^ |
| GA40 | 88% positive, 9% neutral, 3% negative | 22.82 | Heberlein^1^ |
| mtwist | 82% positive, 18% neutral | 34.58 | Astro-temple^2^ |
| twist | 82% positive, 15% neutral, 3% negative | 29.70 | Astro-temple^2^ |
| wave-0 | 94% positive, 3% negative, 3% neutral | 20.82 | Vanrie^3^ |
| Negative | | | |
| CF27 | 94% negative, 6% positive | 28.21 | Heberlein^1^ |
| 21 | 82% negative, 15% neutral, 3% positive | 26.30 | Heberlein^1^ |
| CF31 | 82% negative, 15% neutral, 3% positive | 30.91 | Heberlein^1^ |
| I_am_angry_F | 82% negative, 15% neutral, 3% positive | 34.06 | Manera^4^ |
| rtfrontam | 82% negative, 18% neutral | 32.94 | Astro-temple^2^ |
| Neutral | | | |
| 36 | 91% neutral, 6% negative, 3% positive | 25.79 | Heberlein^1^ |
| F-climbstairs | 82% neutral, 15% positive, 3% negative | 21.49 | Lapenta^5^ |
| sit_down_M | 82% neutral, 12% negative, 3% positive | 30.43 | Manera^4^ |
| spade-90 | 85% neutral, 9% positive, 6% negative | 26.30 | Vanrie^3^ |
| (stand_up_M | 82% neutral, 15% positive, 3% negative | 27.58 | Manera^4^ |
| Ambiguous | | | |
| 22 | 52% neutral, 42% negative, 6% positive | 50.36 | Heberlein^1^ |
| 24 | 61% neutral, 27% negative, 12% positive | 51.24 | Heberlein^1^ |
| 34 | 55% neutral, 42% negative, 3% positive | 43.03 | Heberlein^1^ |
| 45 | 63% neutral, 33% negative, 3% positive | 56.52 | Heberlein^1^ |
| 5 | 42% negative, 42% neutral, 15% positive | 48.27 | Heberlein^1^ |
| frisbeethrow | 52% negative, 30% neutral, 12% positive | 42.58 | Astro-temple^2^ |
| get_down_M | 67% neutral, 27% negative, 6% positive | 50.88 | Manera^4^ |
| M-cheer | 52% positive, 36% neutral, 12% negative | 45 | Lapenta^5^ |
| Mow-0 | 73% neutral, 15% negative, 12% positive | 44.85 | Vanrie^3^ |
| pump-90 | 73% neutral, 18% positive, 14% negative | 47.06 | Vanrie^3^ |
| 27 | 61% negative, 36% neutral, 3% positive | 46.18 | Heberlein^1^ |
| I_am_angry_M | 61% negative, 36% neutral, 3% positive | 45.27 | Manera^4^ |
| ithookam | 55% negative, 42% neutral, 3% positive | 45.12 | Astro-temple^2^ |
| no_F_125 | 55% neutral, 33% negative, 12% positive | 54.85 | Manera^4^ |
| push2 | 67% neutral, 21% negative, 12% positive | 49.21 | Astro-temple^2^ |

*Note*. All scrambled images were taken from Alaerts et al. (2011). Images used were: scramK2_F; scramK7_M; scramK9_F; scramP2_M; scramP4_F. Scrambled images were not included in the pilot study.

1. Available at author’s request (Heberlein et al., 2004)
2. Available at [http://**astro**.**temple**.edu/~tshipley/mocap.html](http://astro.temple.edu/~tshipley/mocap.html)
3. Available at <https://link.springer.com/article/10.3758/BF03206542#SecESM1>
4. Available at <https://link.springer.com/article/10.3758/BRM.42.1.168#SecESM1>
5. Available at author’s request (Lapenta et al., 2017)

**Table S2**

*First- and second-generation ethnic minority participants’ ethnicity*

|  | **1^st^-generation ethnic minority migrants** | **2^nd^-generation ethnic minority migrants** |
| --- | --- | --- |
| N | 94 | 68 |
| N (%) Black/Black British – African | 7 (13%) | 6 (9%) |
| N (%) Black/Black British – any other | 1 (1%) | 0 |
| N (%) Mixed – White and Black Caribbean | 1 (1%) | 0 |
| N (%) Mixed – White and Black African | 0 | 2 (3%) |
| N (%) Mixed – White and Asian | 1 (1%) | 0 |
| N (%) Any other mixed background | 3 (3%) | 0 |
| N (%) Asian/British Asian – Indian | 25 (27%) | 12 (18%) |
| N (%) Asian/British Asian – Pakistani | 15 (16%) | 14 (21%) |
| N (%) Asian/British Asian – Bangladeshi | 6 (6%) | 15 (22%) |
| N (%) Asian/British Asian – any other | 10 (11%) | 6 (9%) |
| N (%) Chinese | 12 (13%) | 1 (1%) |
| N (%) Arab | 6 (6%) | 6 (9%) |
| N (%) Any other background not stated | 7 (7%) | 6 (9%) |

*Note.* The ethnicity categories are taken from the Office for National Statistics, UK (ONS). All participants in the first-generation White group (n = 68) identified as White Other.

% Indicates proportion in the participant group.

**Figure S1**

*Mean ratings of friendliness and aggressiveness of five PLW types*


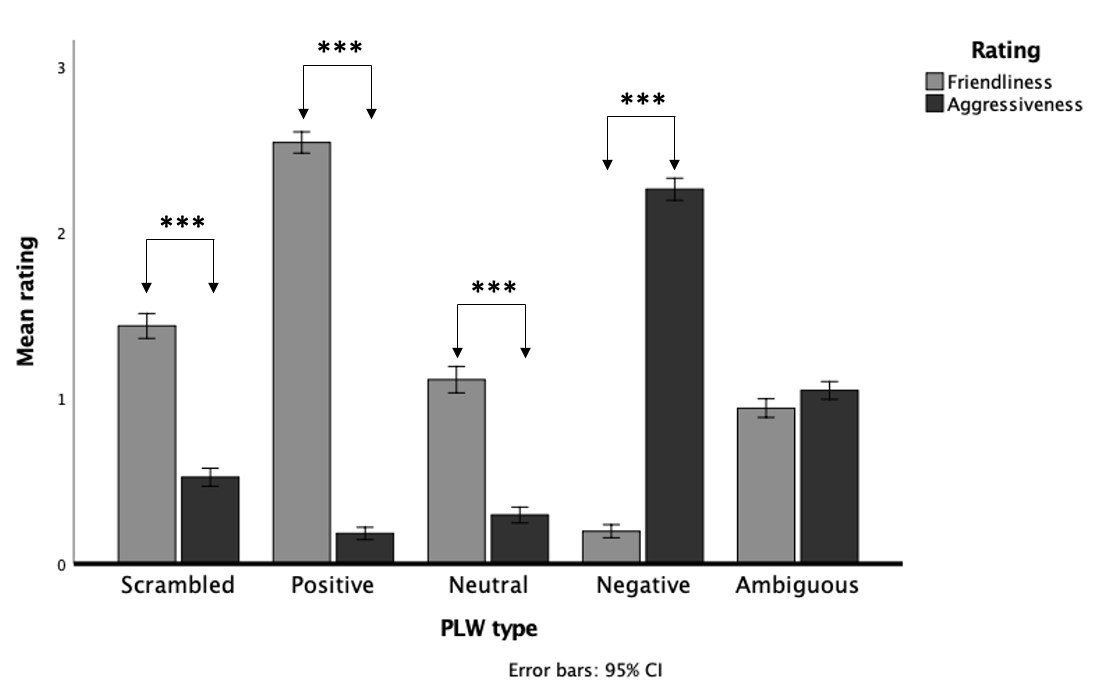


*Note.* Error bars indicate 95% confidence intervals.

*** p < .001
